# Sleep Quality, Depression, and the Risk of Anaemia in Adolescents Aged 10–19 Years During One Year of the COVID‐19 Pandemic in Indonesia

**DOI:** 10.1002/smi.70046

**Published:** 2025-05-03

**Authors:** Muhammad Asrullah, Ahmad Watsiq Maula, Sandra Olivia Frans, Shita Listya Dewi, Monique L'Hoir, Edith J. M. Feskens, Alida Melse‐Boonstra

**Affiliations:** ^1^ Division of Human Nutrition and Health Wageningen University and Research Wageningen the Netherlands; ^2^ Faculty of Medicine, Public Health, and Nursing Centre for Health Policy and Management Universitas Gadjah Mada Yogyakarta Indonesia; ^3^ Department of Biostatistic, Epidemiology, and Population Health Faculty of Medicine, Public Health, and Nursing Universitas Gadjah Mada Yogyakarta Indonesia

**Keywords:** adolescence, depression, haemoglobin concentration, Indonesia, sleep quality

## Abstract

Sleep quality and depression are known to be associated with anaemia in adults, but studies are limited among children and adolescents. The present study aimed to assess the association between sleep quality, depression, and haemoglobin concentration in Indonesian adolescents aged 10–19 years. Data of 452 adolescent boys and girls, aged 10–19 years old, were collected across all subdistricts in Gunungkidul district, Yogyakarta province, Indonesia, in 2021 (baseline) and 2022 (follow‐up). Sleep quality and depression were assesed using The Pittsburgh Sleep Quality Index (PSQI) and The Kessler‐10 Psychological Distress Scale (K10), respectively. Haemoglobin concentration was measured, with corrections applied for altitude and smoking. Anaemia status was defined as haemoglobin < 11.5 g/dL for adolescents aged 10–11 years old, < 12 g/dL for those aged 12–14 years old and for girls aged 15 years and older, and < 13 g/dL for boys aged 15 years old and older. Latent Class Analysis (LCA) was employed to identify distinct subgroups of adolescents based on shared patterns of sleep quality and depression. Multiple linear regression was applied to identify associations between class membership and haemoglobin concentration at baseline and follow‐up, with adjustments for baseline haemoglobin concentration, sex, age, pubertal status, alcohol consumption, smoking status, and household income. The overall prevalence of anaemia was 21% at baseline and 29% at follow‐up, with girls being more affected than boys. LCA yielded 5 classes of sleep quality and depression. We did not find an association between class membership and haemoglobin concentration at baseline. However, in comparison to class A and after adjustments, membership of class B (moderate‐to‐good sleep quality and low risk of depression, with some tiredness) predicted a reduction of 0.43 g/dL (95% CI: −0.79; −0.07), whereas membership of class C (moderate sleep quality and moderate risk of depression) predicted a reduction of 0.49 g/dL (95% CI: −0.94; −0.04) in haemoglobin concentration at 1 year follow‐up. Our study found that poor sleep quality and depression symptoms are associated with lower haemoglobin concentrations over time. Mental health and sleep quality should therefore be considered in intervention programs that address anaemia.

**Summary**

- Studies have demonstrated an increasing prevalence of mental health problems and anaemia among adolescents during the COVID‐19 pandemic. Furthermore, one of the most typical signs of depression in adolescence is a chronic lack of sleep or a severe sleep disorder.
- This study found that the prevalence of anaemia increased from 21% to 29% over the course of 1‐year during the COVID‐19 pandemic in adolescents living in the district of Gunungkidul, Yogyakarta, Indonesia.
- Poor sleep quality and depression did predict lower haemoglobin concentrations 1 year later.
- In addition to existing strategies to prevent and treat anaemia, such as improving diet quality and Iron‐Folic Acid (IFA) supplementation, promoting sleep quality and preventing depression may simultaneously help to reduce the risk to develop anaemia.

## 1 Introduction

Anaemia is a global public health issue impacting an estimated 1.62 billion people worldwide (McLean et al. 2009). Adolescents, especially girls, are vulnerable for anaemia, due to rapid physical growth and pubertal development. Anaemia is the consequence of a low haemoglobin concentration, most commonly due to iron deficiency, which hampers the transport of oxygen throughout the body. In addition to fatigue and poor physical fitness, anaemia may contribute to psychological distress such as moodiness, isolation, feelings of failure, and even depression when left untreated (Azami et al. 2019; Kang et al. 2020; H. S. Lee et al. 2020; Y. J. Lee and Kim 2020). Therefore, anaemia is an important target for intervention in adolescents.

Anaemia has also been linked to changes in sleep patterns, as evidenced in infants (Algarín et al. 2003), children (Uebergang et al. 2017), and adults (Chen‐Edinboro et al. 2018; Jackowska et al. 2013; W.‐H. Kim et al. 2013). Tiredness and apathy, which are common features of anaemia, can as well be the consequence of depression as of poor sleep quality. Conversely, short sleep duration can cause mood disturbances and emotional dysregulation (Baum et al. 2014), while, vice versa, depression can induce poor sleep quality (Oh et al. 2019). Despite these observed associations, the causal relationships between anaemia, depression, and sleep quality remains unclear, raising questions about whether anaemia is a cause, a consequence, or a co‐occurring condition related to these mental health challenges.

Mental disorders are among the most important causes of illness and disability in youth (Abbafati et al. 2020), and are increasing worldwide. The COVID‐19 pandemic has triggered a 25% increase in prevalence of mental health problems globally, particularly among adolescents (World Health Organization 2022). The pandemic has also made an impact on the sleep quality of adolescents, as evidenced by a number of studies (Genta et al. 2021; Meherali et al. 2021; Wang et al. 2022; Zhai et al. 2021). In addition, the pandemic is expected to have increased prevalence of anaemia due to temporary stagnation of health service delivery, for example nutrition education and iron‐folic acid (IFA) supplementation programs through schools and other channels, as well as due to loss of livelihoods and food security (Osendarp 2020).

These global concerns are particularly relevant in the Indonesian context. The prevalence of anaemia was 26.4% in younger adolescents (5–14 years old) and 32% in older adolescents (15–24 years old) in 2018, and had increased since 2013 when it was found to be 17.9% in adolescents aged 13–18 years (Indonesian Ministry of Health 2018). Moreover, the prevalence of depression and other mental health disorders in this age group was reported to be 6.2% and 10%, respectively, in 2018, which is higher than in the general population. In a study among 528 Indonesian adolescents (13–16 years old), it was shown that those with low depression scores experienced a better sleep quality (Setyowati and Chung 2021). Moreover, a study showed that 10.6% of Indonesian students had emotional problems, and 38.1% experienced peer relationship problems during the early phases of the pandemic (Wiguna et al. 2020).

In light of the high and increasing burden of anaemia and mental health challenges among Indonesian adolescents, and the complex interplay between these conditions, we aimed to investigate the longitudinal associations between depressive symptoms, sleep quality and anaemia among Indonesian adolescents aged 10–19 years old. Since poor sleep quality is tightly disentangled with mental health problems (Wesselhoeft et al. 2016), we assessed combined latent classes of sleep quality and depressive symptoms. This study was conducted in 2021–2022, against the backdrop of school closures, social distancing, and hygiene measures due to the COVID‐19 pandemic.

## 2 Methods

### 2.1 Study Setting

The present study has been conducted in the district of Gunungkidul in Yogyakarta Province, Indonesia, where high prevalence of anaemia (23%) and mental health problems (18.4%) were found previously. The total population in Yogyakarta is 3.6 million, of whom 14.5% consists of adolescents aged 10–19 years. In addition, the total population in Gunungkidul district was 758.168 in 2021, of whom 13.1% is aged 10–19 years. The human development index in Gunungkidul was the lowest (70,2) compared to other districts in Yogyakarta. Furthermore, the economic growth rate in Gunungkidul was 5.2% in 2021, with approximately 17.7% of the total population being classified as poor (BPS‐Statistics of Gunungkidul Regency 2022).

### 2.2 Study Design and Data Collection

Data of 576 adolescents (286 boys and girls, respectively) aged 10–19 years were collected across all subdistricts of Gunungkidul district, Yogyakarta province, Indonesia. The required sample size was calculated manually using a general formula for estimating a single population proportion, assuming that the prevalence of anaemia among adolescents in Gunungkidul was similar to the previously reported prevalence for Yogyakarta province (23%) (BPS‐Statistics of Gunungkidul Regency 2022).

$$n=\frac{Z^{2}(p\left(1-p\right)}{d^{2}}$$

A 95%‐confidence level (*Z*) and 5% margin of error (*d*) were used for the calculation. To account for potential loss to follow‐up, a 10% adjustment was applied by increasing the total number of adolescents included. This adjustment was based on previous school‐based cohort studies in similar settings (Craddock et al. 2024; Thomas et al. 2012), where attrition due to factors such as irregular school attendance, relocation, or withdrawal of consent typically ranged from 5% to 15%, making 10% a conservative and reasonable estimate. To produce representative data, a listing survey was performed by visiting all villages and households with adolescents, with a total of 9942 adolescents available for random inclusion. A multi‐stage sampling framework was used. In the first stage, variable numbers of subdistricts were randomly selected from 3 zones (north, central, and south area) using Probability Proportionate to Size sampling, so that more sub‐districts were selected in zones with more subdistricts. In the second stage, two villages were randomly selected from each included subdistrict, and, in the last stage, 8 girls and 8 boys aged 10–14 years, and 8 girls and 8 boys aged 15–19 years were selected from each village by simple random sampling. The survey was conducted in 9 subdistricts and 18 villages across the district. Adolescents who refused to participate were randomly replaced with other adolescents in the same village. However, since the data were collected only in rural areas, they do not represent any urban areas.

In Indonesia, schools were closed due to the COVID‐19 pandemic from March 2020 until April 2022. The first round of data collection (baseline) was conducted from May to August 2021 and was completed just before strict lockdowns were implemented due to the outbreak of the delta variant of the coronavirus. We collected follow‐up data in the period May–July 2022. Before conducting the interviews, all adolescents and their parents/guardians signed an informed consent form. All adolescents received a study kit containing a bag, headset for online classes, and a drinking bottle as remuneration for their participation. In total, complete data from 576 adolescents (288 boys and 288 girls) were collected at baseline. There were 24 adolescents who refused to participate and were subsequently replaced to reach the final sample size. For each refusal, a replacement was randomly selected from the adolescent list in the same village, matching the non‐participant by age and sex to maintain sample comparability. This process was repeated until the required sample size was achieved. Furthermore, only 452 adolescents (222 boys and 230 girls) participated at follow‐up, with most adolescents lost to follow up due to migration. However, characteristics of adolescents did not differ between those in the original sample and those who were lost to follow‐up (21%) (Supporting Information S1).

Twelve data collectors were involved in this study, who received a 2‐day online and a 2‐day offline training to ensure that the same quality standards were applied by all data collectors. The same data collectors were hired and re‐trained for data collection at follow‐up. Data collectors visited all selected adolescents in their home with permission from the head of each subdistrict, village, and sub‐village leader. The interviews were conducted after an information sheet and consent form had been signed by adolescents and their parents/guardians. Two software programs for mobile data collection were employed, the ODK Collect (Get ODK Inc.) and the Offline Surveys (LimeSurvey GmbH) android‐based application. Mobile data collection was set up to minimise error in data entry and handling. The software programs were tested during a pilot phase for practicality and were further evaluated during the first week of data collection. To ensure the quality of data collection, automated checks were built in to prevent inappropriate data entry during the interview. In addition, we also recorded the interviews to enable data verification. If a problem in power supply was encountered, or if the software tools could not be opened either online or offline, data collectors used a paper‐based questionnaire and processed the data the same day into the online database, under supervision of the field coordinator and data managers. After data processing, the paper‐based questionnaires were uploaded to the ODK software tool to allow any further data verification if required. Every data manager was responsible for data verification of four data collectors. Regarding data quality, we randomly re‐visited several respondents in each village. Questions were asked to respondents and heads of villages to validate the interview process, the listing survey, and the mapping methods by the data collectors. Data verification was done by data managers together with the data collectors every afternoon after data collection, in which the consistency, completeness, and the logic of the answers were checked. In case any missing information or inconsistencies were found, the data collectors were asked to contact or re‐visit the respondents for clarification the day after, in order to obtain the required information without a time bias. Furthermore, adolescents were interviewed by the same data collectors during baseline and follow‐up in order to minimise interview bias. The interviews were conducted outside the home without presence of the parents to maintain objectivity. Interviews took up to 90 min to complete.

### 2.3 Study Variables

All variables were assessed both at baseline and follow‐up. For the present analysis haemoglobin concentration at follow‐up was the outcome variable, and measurements at baseline were considered as determinants or confounders.

### 2.4 Assessment of Anaemia, Sleep Quality, and Depression

Haemoglobin data were collected by HemoCue 201+ (HemoCue AB, Ängelholm, Sweden) from all adolescents who were willing to have their finger prick blood taken. The HemoCue 201+ was calibrated shortly before the study started by the Certified Health Laboratory and Calibration Unit, and was checked by data collectors before each measurement. Before each measurement, data collectors checked whether the device was functioning properly by ensuring it was turned on and did not display any error messages. After correction of haemoglobin concentrations for altitude and smoking (World Health Organization 2011), anaemia was classified as haemoglobin < 11.5 g/dL for adolescents aged 10–11 years old, < 12 g/dL for those aged 12–14 years old and for girls aged 15 years and older, and < 13 g/dL for boys aged 15 years old and older. All adolescents included at baseline (*n* = 576) and follow‐up (*n* = 452) underwent the finger prick test.

Sleep quality was assessed using The Pittsburgh Sleep Quality Index (PSQI) which is a self‐reported questionnaire that evaluates sleep quality and disruptions over a 1‐month period (Supporting Information S1). Subjective sleep quality, sleep latency, sleep length, habitual sleep performance, sleep disturbances, use of sleeping medication, and daytime dysfunction are among the 19 items which together form seven ‘component’ ratings that each weighted equally on a 0–3‐point scale, with increasing scores ranging between 0 and 21 indicating worse sleep quality. The PSQI has been validated for adolescents in the Indonesian setting with high reliability and validity (Cronbach's alpha of 0.72 and total‐item correlations of 0.36–0.56) (Setyowati and Chung 2021).

The Kessler‐10 Psychological Distress Scale (K10) was used to measure non‐specific psychological distress in the anxiety‐depression spectrum, that is, levels of nervousness, agitation, psychological fatigue, and depression in the past 4 weeks. It has been translated and validated previously in Bahasa Indonesia (all the scales had Cronbach's alpha > 0.8, with sensitivity of 85.7% and specificity of 74.7%). The K10 comprises of 10 questions that have strong psychometric properties by which psychiatric cases and non‐psychiatric cases can be discriminated (Supporting Information S1). A cut‐off ≥ 18 (scoring range: 10–50) was used to determine the prevalence of depression among adolescent boys and girls (Tran et al. 2019).

### 2.5 Assessment of Potential Covariates

A broad range of potential covariates was assessed, both at the individual and at the household level. At the individual level, physical activity was measured with the Physical Activity Questionnaire for all adolescents (PAQ‐A), which is an activity checklist that asks for participation in different types of activities and sports, efforts during physical education classes, and activity during lunch, after school, in the evening and in the weekend during the past 7 days (Kowalski et al. 2004). Weight and height were measured to assess nutritional status. Anthropometric measurements were mostly taken in the respondents' home straight after the interviews, to minimise response bias. Adolescent height was measured to the nearest 0.1 cm using a stadiometer (Kenko Stadiometer 250, China). Weight was measured without shoes and other accessories and recorded to the nearest 0.1 kg using a flat digital weighing scale (GEA EB1622, Indonesia), placed on a flat surface. Height and weight were measured and recorded twice, with an acceptable difference of 0.2 cm and 0.1 kg. All anthropometric equipment was calibrated by the Certified Health Laboratory and Calibration Unit in Yogyakarta. Before each measurement, data collectors checked that the equipment was functioning correctly and ready for use.

Presence of eating disorders was assessed using the EAT‐26 questionnaire, based on 26 items, which has been used previously in Indonesia (Sulistyan et al. 2016). Body image perception and self‐image perception were assessed using the Figure Rating Scale (FRS) (Stunkard et al. 1983), as also used in an earlier Indonesian study among adolescent girls and boys (Sari et al. 2020). The FRS is gender‐specific, with a scale that ranges from 1 (thinnest) to 9 (largest). Food consumption was assessed by recalling the consumption of ten food groups during the last 24 h, as per the Minimum Dietary Diversity for Women (MDD‐W): starchy staples, dark‐green leafy vegetables, vitamin A‐rich fruits and vegetables, other vegetables, other fruits, flesh and organ meat, eggs, fish, legumes/nuts/seeds, and milk products (FAO and & USAID 2016). Dietary diversity score (DDS) was assessed using the total count out of the ten food groups. We also assessed the consumption of risky foods, referring to the consumption of salty food, high‐fat food, and soft drinks, which was classified as low if adolescents consumed such foods only 1–2 times a week, moderate if they consumed them 3–6 times a week, and high if they consumed them every day. Smoking status and alcohol consumption were assessed using questions on history of cigarette smoking and alcohol consumption on a daily and weekly basis.

Pubertal stage was assessed by the Picture‐Based Interview about Puberty (PBIP) (Carskadon and Acebo 1993; Shirtcliff et al. 2009). The 5‐stage PBIP was employed as a self‐administered questionnaire, which means that the respondents had to select the best‐matching picture which represented their stage of pubertal development by themselves. Adolescents were classified into pre‐pubertal (stage 1), early pubertal (stage 2 or 3), and late pubertal (stage 4 or 5). Self‐aspiration for higher education was assessed by exploring adolescents' desired level of education in an ideal, unconstrained scenario, as done in the Young Lives Study (https://www.younglives.org). A binary yes/no variable was constructed to represent high aspirations, defined as the goal of attaining high school and university‐level education.

At the household level, parental education was defined as the highest education level attained by parents. In addition, parental occupation was assessed, defined as the daily activities in which parents engage to generate income. Household size was assessed as the total number of individuals who lived in the same house building, without considering the household's registration. This number is therefore not limited to one household only, because it is possible in Indonesia to have more than one households in the same house building. Household total income was defined as the total income from all family members for food and non‐food expenses and classified as less than Rp 3.000.000 (low), between Rp 3.000.000 and Rp 5.000.000 (moderate), and more than Rp 5.000.000 (high). Household food access was measured based on purchasing behaviour regarding ten food groups before the pandemic (6 months before) and during the pandemic (6 months after the pandemic started). For each time point, each food item was scored as 1 when frequently purchased (maximum score = 10). The sum provided the basis for categorising household food access by dividing all scores to tertiles (3 categories: high, moderate, and low). A more detailed explanation of all the variables can be found in Supporting Information S1.

### 2.6 Statistical Analysis

All questionnaires and results were checked for completeness and consistency of responses. Age of adolescents was classified into the categories 10–≤ 13 years, > 13–≤ 16 years, > 16–≤ 18 years and > 18–19 years. Occupation of adolescents was classified as ‘student’, ‘work’, and ‘unemployed’. Descriptive statistics, adjusted for sampling weights that were calculated based on sex and age group per village, were used to produce prevalence characteristics among study participants. Subsequently, latent classes of sleep and depressive symptoms were constructed, and linear regression analysis was used to assess associations between the resulting classes and Hb at baseline and follow‐up (Bakk et al. 2013).

A latent class model was built using the bias‐adjusted 3‐step approach in Latent‐Gold (version 5.1, Statistical Innovations Inc., Tilburg, the Netherlands) with cluster combinations for sleep and depression. Indicator variables were the 7 components of the sleep quality scale and the 10 components of the depression scale, entered as ordinal‐fixed variables, indicating scores 0–3 for each sleep quality item (maximum score: 21) and scores 0–4 for each depression item (maximum score: 40). Models with 1–7 classes were tested and compared based on their bayesian information criteria (BIC), akaike's information criteria (AIC), the number of parameters (Npar), and bivariate residual (BVR) values, as recommended (Vermunt and Magidson 2016). The Wald‐test was used to evaluate significance of variables to the latent class formation. After deciding on the optimal number of classes, direct effects were adjusted for one by one, starting with the largest direct effect until there was no residual association left as indicated by a BVR value ≤ 3.84 (Van Kollenburg et al. 2015; Vermunt and Magidson 2016). The individuals were then classified into the latent classes. Thereafter, the relationship between the latent classes and anaemia at baseline and at follow‐up was investigated using six hierarchical linear regression models, adjusted for variables at adolescent level (sex, age, pubertal status, alcohol use and smoking) and at household level (household income), which were identified amongst other factors a priori based on previous literature (Azami et al. 2019; Kang et al. 2020; Y. J. Lee and Kim 2020).

## 3 Results

### 3.1 General Characteristics

The overall prevalence of anaemia was 21% at baseline and 29% at follow‐up, with girls being more affected than boys (31% vs. 9% at baseline, and 42% vs. 15% at follow‐up, respectively). The average AAM in adolescent girls was 12.5 years (SD: 1.2). Depression occurred in approximately 28% of adolescent boys and girls at baseline, with higher prevalence among girls (35%) than among boys (21%).

### 3.2 Latent Classes of Sleep Quality and Depression

**FIGURE 1:**
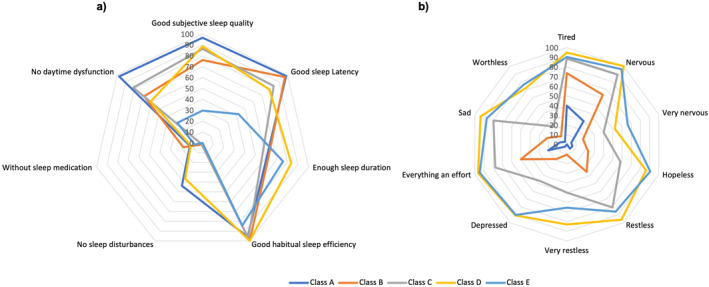
(a) Probability of sleep quality scores among latent classes A–E; and (b) Probability of depression scores among latent classes A–E. Class A: (48.3%), with good sleep quality and low risk of depression; Class B: (22%), with moderate‐to‐good sleep quality and low risk of depression, but with some tiredness; Class C: (17%), with moderate sleep quality and moderate risk of depression; Class D: (8%), with moderate sleep quality and high risk of depression; and Class E: (4.7%), with poor sleep quality and high risk of depression.

LCA analysis yielded five classes of sleep quality and depression (Supporting Information S1): Class A (48.3%), with good sleep quality and low risk of depression; Class B (22%), with moderate‐to‐good sleep quality and low risk of depression, but with some tiredness; Class C (17%), with moderate sleep quality and moderate risk of depression; Class D (8%), with moderate sleep quality and high risk of depression; and Class E (5%), with poor sleep quality and high risk of depression (see Figure 1). Further demographic and behavioural characteristics of adolescents within each class are described in Table 1 and elaborated in the Discussion.

**TABLE 1:** Characteristics of the adolescents (*N* = 452) based on latent classes of sleep quality and depression.

Characteristics	Latent class, *n* (%)^a^					*p*‐value
	Class A	Class B	Class C	Class D	Class E	
	*N* = 203 (48%)	*N* = 119 (22%)	*N* = 64 (17%)	*N* = 44 (8.0%)	*N* = 22 (4.7%)	
Adolescent characteristics						
Sex						< 0.001
Boys, *n* (%)	122 (60.1)	58 (48.7)	19 (26.7)	21 (47.7)	2 (9.1)	
Girls, *n* (%)	81 (39.9)	61 (51.3)	45 (70.3)	23 (52.27)	20 (90.9)	
Age, y (mean ± SD)	13.6 ± 2.6	14.7 ± 2.6	15.3 ± 2.1	13.3 ± 2.2	15.2 ± 1.8	< 0.001
Age group						< 0.001
10 to ≤ 13 years	75 (37.0)	28 (23.5)	6 (9.4)	19 (43.2)	2 (9.2)	
> 13– ≤ 16 years	75 (37.0)	43 (36.1)	25 (39.1)	16 (36.4)	10 (45.4)	
> 16– ≤ 18 years	44 (21.6)	39 (32.8)	31 (48.4)	9 (20.4)	10 (45.4)	
> 18–19 years	9 (4.4)	9 (7.6)	2 (3.1)	0 (0)	0 (0)	
Adolescent occupation, *n* (%)						0.242
Unemployed	17 (8.4)	14 (11.8)	6 (9.4)	1 (2.3)	0 (0)	
Student	181 (89.1)	99 (83.2)	57 (89.1)	43 (97.7)	21 (95.5)	
Work	5 (2.5)	6 (5.0)	1 (1.5)	0 (0)	1 (4.5)	
Physical activity, *n* (%)						0.517
Low	64 (31.5)	44 (37.0)	22 (34.4)	10 (22.7)	10 (45.5)	
Moderate	81 (39.9)	46 (38.6)	29 (45.3)	18 (40.9)	7 (31.8)	
High	58 (28.5)	29 (24.4)	13 (20.3)	16 (36.4)	5 (22.7)	
Nutritional status (BMI *z*‐score), *n* (%)						0.812
Severely thin	3 (1.5)	4 (3.4)	1 (1.6)	0 (0)	0 (0)	
Thin	18 (9.0)	13 (10.9)	4 (6.3)	5 (11.6)	3 (13.6)	
Normal	128 (63.7)	79 (66.4)	49 (76.5)	28 (65.2)	16 (72.7)	
Overweight	32 1(5.9)	16 (13.4)	7 (10.9)	5 (11.6)	2 (9.1)	
Obese	20 (9.9)	7 (5.9)	3 (4.7)	5 (11.6)	1 (4.5)	
Anaemia at baseline (yes), *n* (%)	45 (22.2)	29 (24.4)	24 (37.5)	7 (15.9)	6 (27.3)	0.082
Haemoglobin at baseline (mean ± SD)	13.3 ± 1.8	13.1 ± 1.6	12.8 ± 1.7	12.8 ± 1.5	12.6 ± 1.7	0.248
Anaemia at follow‐up (yes), *n* (%)	52 (25.6)	41 (34.5)	29 (45.3)	15 (34.1)	10 (45.5)	0.025
Haemoglobin at follow‐up (mean ± SD)	13.2 ± 1.8	12.7 ± 1.7	12.4 ± 2.0	12.7 ± 1.7	12.3 ± 1.6	< 0.001
Eating disorder (yes), *n* (%)	5 (2.5)	4 (3.4)	2 (3.12)	2 (3.12)	1 (4.6)	0.944
Diet diversity (inadequate), *n* (%)	15 (7.4)	7 (5.9)	2 (3.1)	3 (6.8)	2 (9.1)	0.772
Diet diversity scores, (mean ± SD)	8.5 ± 2.4	8.7 ± 2.0	8.9 ± 1.6	8.6 ± 2.2	8.3 ± 2.7	0.790
Risk consumption						0.448
High	157 (77.4)	85 (71.4)	44 (68.7)	35 (79.5)	17 (72.3)	
Moderate	24 (11.8)	15 (12.6)	6 (9.4)	6 (13.6)	2 (9.1)	
Low	22 (10.8)	19 (16.0)	14 (21.9)	3 (6.8)	3 (13.6)	
Smoking status (yes), *n* (%)	29 (14.3)	28 (23.5)	11 (17.2)	2 (4.6)	4 (18.2)	0.046
Alcohol consumption (yes), *n* (%)	2 (1.0)	4 (3.4)	2 (3.1)	0 (0.0)	2 (9.1)	0.085
Pubertal status (based on PBIP), *n* (%)						< 0.001
Pre‐pubertal	59 (29.1)	18 (15.1)	4 (6.2)	14 (31.8)	2 (9.1)	
Early pubertal	115 (56.6)	81 (68.1)	41 (64.1)	23 (52.3)	15 (68.2)	
Late pubertal	229 (14.3)	20 (16.8)	19 (29.7)	7 (15.9)	5 (22.7)	
Self‐aspiration for higher education, *n* (%)	82 (42.4)	55 (46.2)	32 (50.0)	27 (61.4)	14 (63.6)	0.013
Household characteristic						
Paternal education, *n* (%)						0.436
No education	17 (8.4)	11 (9.2)	5 (7.8)	1 (2.3)	1 (4.5)	
Elementary school	121 (59.9)	78 (65.5)	35 (54.7)	28 (65.1)	9 (40.9)	
Senior high school	57 (28.2)	25 (21.0)	20 (31.2)	13 (30.2)	10 (45.5)	
University	7 (3.5)	5 (4.3)	4 (6.2)	1 (2.3)	2 (9.1)	
Maternal education, *n* (%)						0.175
No education	18 (9.1)	10 (8.4)	6 (9.4)	1 (2.3)	1 (4.8)	
Elementary school	129 (65.5)	76 (64.4)	39 (60.9)	31 (72.1)	10 (47.6)	
Senior high school	40 (20.3)	27 (22.8)	15 (23.4)	8 (18.6)	5 (23.8)	
University	10 (5.1)	5 (4.2)	4 (6.3)	3 (7.0)	5 (23.8)	
Paternal occupation, *n* (%)						< 0.001
Unemployed	0 (0)	0 (0)	0 (0)	0 (0)	1 (4.8)	
Unsecured job	89 (45.6)	60 (52.6)	23 (39.7)	18 (42.9)	5 (23.8)	
Secured job	106 (54.4)	54 (47.4)	35 (60.3)	24 (57.1)	15 (71.4)	
Maternal occupation, *n* (%)						0.594
Unemployed	70 (36.5)	36 (34.3)	22 (40.0)	13 (30.9)	5 (23.8)	
Unsecured job	95 (49.5)	48 (45.7)	24 (43.6)	24 (5.2)	10 (47.6)	
Secured job	27 (14.0)	21 (20.0)	9 (16.4)	5 (11.9)	6 (28.6)	
Number of household members > 5 people, *n* (%)	43 (21.2)	24 (20.2)	16 (25)	9 (20.5)	3 (13.6)	0.845
Household income, *n* (%)						0.016
Low	169 (83.3)	101 (84.9)	53 (82.8)	37 (84.1)	15 (68.2)	
Moderate	31 (15.3)	15 (12.6)	8 (12.5)	5 (11.4)	3 (13.6)	
High	3 (1.4)	3 (2.5)	3 (4.7)	2 (4.6)	4 (18.2)	
Household food access at baseline, *n* (%)						
Before pandemic						0.271
High	59 (39.1)	41 (40.6)	14 (25.9)	15 (40.5)	3 (17.6)	
Moderate	56 (37.1)	36 (35.6)	23 (42.6)	9 (24.3)	7 (41.2)	
Low	36 (23.8)	24 (23.8)	17 (31.5)	13 (35.2)	7 (41.2)	
After pandemic						0.148
High	77 (51.3)	46 (44.7)	27 (52.9)	18 (51.4)	5 (29.4)	
Moderate	48 (32.0)	41 (39.8)	14 (27.5)	6 (17.1)	7 (41.2)	
Low	25 (16.7)	16 (15.5)	10 (19.6)	11 (31.5)	5 (29.4)	

^a^ Class A: Good sleep quality and low risk of depression; Class B: Moderate‐to‐good sleep quality and low risk of depression, with some tiredness; Class C: Moderate sleep quality and moderate risk of depression; Class D: Moderate sleep quality and high risk of depression; Class E: Poor sleep quality and high risk of depression.

### 3.3 Regression Analyses

No association was found between latent class membership and haemoglobin concentration at baseline in crude and adjusted models (Table 2). However, we found inverse associations between membership of latent classes B, C and E at baseline, and haemoglobin concentration at follow‐up (Table 3). These associations largely remained after adjusting for haemoglobin at baseline, sex, pubertal status, alcohol consumption, smoking status, and household income (Table 3, model 7). We found adolescent sex to be a significant moderator of the association between latent class membership and haemoglobin concentration at follow‐up (Model 2), with the association being weaker among girls compared to boys. Meanwhile, after additionally adjusting for age and pubertal status (model 3 and 4), the associations became somewhat stronger again. The final model (Model 7) demonstrated that, compared to Class A, membership in Class B (moderate‐to‐good sleep quality and low risk of depression, with some tiredness) and Class C (moderate sleep quality and moderate risk of depression) was significantly associated with reductions in haemoglobin concentration at 1‐year follow‐up by 0.43 g/dL (95% CI: −0.79; −0.07) and 0.49 g/dL (95% CI: −0.94; −0.04), respectively. The model showed moderate levels of psychological distress and sleep disturbances can have measurable effects on physiological health, such as reduced haemoglobin levels.

**TABLE 2:** Linear regression analysis of sleep quality index and depression with baseline haemoglobin concentration (*N* = 452).

Statistical model	Class A (*N* = 203)	Haemoglobin (g/dL) at baseline											
		Class B (*N* = 119)			Class C (*N* = 64)			Class D (*N* = 44)			Class E (*N* = 22)		
		*β*	SE	(95% CI)	*β*	SE	(95% CI)	*β*	SE	(95% CI)	*β*	SE	(95% CI)
Model 1 (crude)	Ref.	−0.13	0.20	−0.53; 0.26	−0.42	0.25	−0.91; 0.08	−0.36	0.29	−0.94; 0.20	−0.66	0.39	−1.44; 0.11
Model 2	Ref.	0.03	0.19	−0.60; 0.34	0.03	0.24	−0.41; 0.50	−0.19	0.26	−0.71; 0.34	0.09	0.37	−0.64; 0.82
Model 3	Ref.	−0.07	0.24	−0.60; 0.34	−0.13	0.24	−0.60; 0.34	−0.15	0.26	−0.68; 0.36	−0.05	0.37	−0.78; 0.67
Model 4	Ref.	−0.03	0.18	−0.41; 0.34	−0.09	0.24	−0.56; 0.38	−0.17	0.27	−0.70; 0.3	−0.01	0.37	−0.73; 0.72
Model 5	Ref.	−0.11	0.18	−0.47; 0.27	−0.16	0.24	−0.66; 0.39	−0.14	0.27	−0.68; 0.38	−0.13	0.37	−0.87; 0.60
Model 6	Ref.	−0.10	0.19	−0.48; 0.27	−0.14	0.24	−0.61; 0.33	−0.13	0.26	−0.66; 0.39	−0.06	0.38	−0.81; 0.69

*Note:* Class A: good sleep quality and low risk of depression; Class B: moderate‐to‐good sleep quality and low risk of depression, with some tiredness; Class C: moderate sleep quality and moderate risk of depression; Class D: moderate sleep quality and high risk of depression; Class E: poor sleep quality and high risk of depression. Model 1: crude model; *R* squared: 0.0120. Model 2: adjusted for sex; *R* squared: 0.1713. Model 3: adjusted for sex and age; *R* squared: 0.1897. Model 4: adjusted for sex and pubertal status; *R* squared: 0.1865. Model 5: model 4, additionally adjusted for alcohol use and smoking; *R* squared: 0.2006. Model 6: model 5, additionally adjusted for household income; *R* squared: 0.2021.

**TABLE 3:** Linear regression analysis of sleep quality index and depression with haemoglobin concentration at 1 year follow‐up (*N* = 452).

Statistical model	Class A (*N* = 203)	Haemoglobin (g/dL) at follow‐up											
		Class B (*N* = 119)			Class C (*N* = 64)			Class D (*N* = 44)			Class E (*N* = 22)		
		*β*	SE	(95% CI)	*β*	SE	(95% CI)	*β*	SE	(95% CI)	*β*	SE	(95% CI)
Model 1 (crude)	Ref.	**−0.50**	0.21	−0.92; −0.08	**−0.83**	0.27	−1.35; −0.32	−0.46	0.30	−1.06; 0.14	**−0.87**	0.36	−1.69; −0.06
Model 2	Ref.	**−0.44**	0.19	−0.81; −0.07	**−0.63**	0.23	−1.09; −0.17	−0.28	0.30	−0.81; 0.26	**−0.54**	0.36	−1.26; −0.18
Model 3	Ref.	−0.32	0.18	−0.66; 0.04	−0.32	0.23	−0.76; 0.12	0.18	0.26	−0.68; 0.33	−0.02	0.36	−0.72; 0.68
Model 4	Ref.	**−0.36**	0.18	−0.72; −0.01	−0.40	0.23	−0.85; 0.04	0.16	0.25	−0.67; 0.33	−0.09	0.35	−0.79; 0.60
Model 5	Ref.	**−0.38**	0.18	−0.74; −0.03	−0.42	0.23	−0.87; 0.03	−0.17	0.26	−0.67; 0.33	−0.11	0.36	−0.80; 0.60
Model 6	Ref.	**−0.42**	0.18	−0.77; −0.06	**−0.47**	0.23	−0.91; −0.16	−0.16	0.25	−0.67; 0.34	−0.25	0.35	−0.96; 0.45
Model 7	Ref.	**−0.43**	0.18	−0.79; −0.07	**−0.49**	0.23	−0.94; −0.04	−0.18	0.25	−0.67; 0.33	−0.35	0.36	−1.07; 0.36

*Note:* Class A: good sleep quality and low risk of depression; Class B: moderate‐to‐good sleep quality and low risk of depression, with some tiredness; Class C: moderate sleep quality and moderate risk of depression; Class D: moderate sleep quality and high risk of depression; Class E: poor sleep quality and high risk of depression. Model 1: crude model; *R* squared: 0.0313. Model 2: adjusted for haemoglobin at baseline; *R* squared: 0.2490. Model 3: adjusted for haemoglobin at baseline and sex; *R* squared: 0.3277. Model 4: adjusted for haemoglobin at baseline, sex, and age; *R* squared: 0.3314. Model 5: model 3, additionally adjusted for pubertal status; *R* squared: 0.3379. Model 6: model 4, additionally adjusted for alcohol use and smoking; *R* squared: 0.3499. Model 7: model 5, additionally adjusted for household income; *R* squared: 0.3. Bold values indicate statistically significant result based on 95% Confidence Interval.

## 4 Discussion

In this analysis, we examined the association between combined latent classes of sleep quality and depression and haemoglobin concentration at baseline (2021) and at 1 year follow‐up (2022) in Indonesian adolescents. We found that the prevalence of anaemia had increased by 8% points (from 21% to 29%) over the course of 1 year, with girls more likely to be anaemic than boys. Furthermore, we did not find an association between latent class membership and haemoglobin concentrations at baseline. However, after controlling for baseline haemoglobin concentration and other covariates, and relative to latent class A (those with good sleep quality and low risk of depression), class membership predicted a reduction of 0.43 g/dL (95% CI: −0.79; −0.07) and 0.49 g/dL (95% CI: −0.94; −0.04) in haemoglobin concentration at follow‐up for those with moderate‐to‐good sleep quality and low risk of depression, with some tiredness (class B), and for those with moderate sleep quality and moderate risk of depression (class C), respectively.

The lack of association between haemoglobin concentration and depression at baseline aligns with prior research. We found previously that anaemia and age at menarche (AAM) were not cross‐sectionally linked to common mental disorders (CMD) among Indonesian adolescents. However, haemoglobin concentration was positively associated with CMD when combined with asthma and smoking (Asrullah et al. 2022). This suggests that comorbidities or interactions between physical and mental health conditions may be important contributors to haemoglobin variation. In contrast, a study in Mexican adolescents found that iron‐deficient girls were more likely to experience depressive symptoms, with low haemoglobin concentrations and higher body weight (Zarate‐Ortiz et al. 2022).

Regarding the lack of association between baseline haemoglobin concentration and sleep quality, a study in young Indonesian women (15–24 years) also found no significant relationship between anaemia and sleep disturbances (Helmyati et al. 2023). However, other evidence suggests that iron deficiency does contribute to impaired sleep quality, including restless sleep and other sleep disorders (Kordas et al. 2007; Leung et al. 2020). Additionally, short sleep duration has previously been linked to poor food quality, increased consumption of soft drinks and stimulant beverages before bedtime, and micronutrient deficiencies (Hermes et al. 2022), which could impact haemoglobin concentrations and overall health. Shared biological mechanisms, such as the MEIS1 gene, which is linked to both anaemia and sleep disturbances (Hammerschlag et al. 2017), or altered brain blood flow associated with sleep disturbances and anaemia (Jackowska et al. 2013; Spira et al. 2016), may explain these associations. However, we did not explore such biological mechanisms in this study. Differences in findings between studies may also stem from contextual factors such as population characteristics, cultural practices, or differences in measurement tools and time frames.

Despite the lack of associations at baseline, we did find inverse associations between membership of latent classes B, C, and E at baseline and haemoglobin concentration at follow‐up, after adjusting for baseline haemoglobin, sex, pubertal status, alcohol consumption, smoking, and household income. These findings are not in line with a previous 8‐year prospective cohort study among 2920 adults in the Netherlands, which did not show an independent association between depression, including poor sleep symptoms, and haemoglobin or anaemia status (Lever‐van Milligen et al. 2014). Also, a previous study with 1 year follow‐up showed that sleep disturbances at baseline did not impact anaemia, although it did impact depressive symptoms and overall physical functioning (Urrila et al. 2014). However, a prospective study of similar duration among 84,791 Chinese people indicated that both short as well as long sleep duration were associated with increased risk of anaemia (Liu et al. 2018). Also, a longitudinal study of 6465 men and women aged 50–99 years found that short sleep duration and sleep disturbances were associated with low haemoglobin concentrations (men: OR: 1.73, C.I. 1.13–2.65; women: OR: 1.59, C.I. 1.02–2.46) (Jackowska et al. 2013). Moreover, anaemic high‐school boys and girls in Norway were found to be more at risk of insomnia‐related depression (Langvik et al. 2019). These inconsistencies suggest that developmental stage, cultural context, and follow‐up duration may play critical roles in the observed relationships.

To better understand why these associations may exist, it is important to consider both biological and behavioural mechanisms. Explanations can be sought in behavioural aspects, such as poor dietary intake. Altered appetite due to sleep disturbances and depression can lead to unhealthy food choices, lower nutrient intake, and reduced haemoglobin concentrations (Hermes et al. 2022; Landis et al. 2009; Yau and Potenza 2013). Depression‐related symptoms such as low energy and lack of interest may also reduce the effort to prepare healthy meals and impair diet quality (Gibson‐Smith et al. 2018; Leigh Gibson 2006). Additionally, hyperactivity of the hypothalamic‐pituitary‐adrenal (HPA) axis in depression and chronic stress increases glucocorticoid levels, thereby stimulating cravings for calorie‐dense but nutrient‐poor foods (Pecoraro et al. 2004). These factors collectively highlight how depression and poor sleep can negatively affect diet and haemoglobin levels. Moreover, disrupted circadian rhythms may impair iron metabolism and erythropoiesis, potentially lowering haemoglobin concentration.

Our findings further showed that individuals in class B, C, and E more frequently engaged in smoking and alcohol consumption. Adjusting for substance use in our analysis highlighted a stronger negative association between class membership and haemoglobin concentrations and emphasised the significant influence of these lifestyle factors. For instance, smoking increases the risk of hypoxia and chronic shortness of breath, which can lead to poorer sleep quality and, over time, contribute to stress and anxiety (Anandha Lakshmi et al. 2014). Moreover, the relationship between alcohol consumption, sleep disturbances, and depression is well‐documented (Robillard et al. 2021; Musse et al. 2020). Similarly, in the U.S.A, moderate to severe depression was linked to both increased alcohol use and worsening sleep quality during stay‐at‐home orders due to COVID‐19 (Knell et al. 2020). Adding to this, a study of 21,440 Korean adults found that men who began drinking before the age of 19 years had a higher likelihood of depressive symptoms, while women who consumed seven or more drinks per occasion also had higher odds of depression, especially when their sleep duration was below average (Y. Kim et al. 2024). Hence, smoking and alcohol consumption appear to be related to both poorer sleep quality and depression, as confirmed in our study. These variables were considered as confounders in our analysis, rather than effect modifiers, and their inclusion in the regression models strengthened the observed associations.

We noted that girls were overrepresented in latent classes C and E, which were two of the more problematic sleep‐depression clusters. Anaemia was also more prevalent in both of these classes, at baseline as well as at follow‐up. In addition, these classes tended to represent adolescents at a later pubertal stage. Previous studies have found differences in sleep disturbances in association with onset of menses in adolescent girls (Johnson et al. 2006; Knutson 2005; Scholle et al. 2011). Furthermore, risk of anaemia can be expected to be higher in older girls due to the longer exposure time to menses (Sekhar et al. 2016). As shown in our regression models, pubertal development indeed explained part of the variance, after adjustment for baseline haemoglobin concentration, age, and sex.

The overall increase in prevalence of anaemia from baseline to follow‐up which we found may also be due to the COVID‐19 related school closures and lockdowns. During the pandemic, the Weekly Iron‐Folic Acid Supplementation (WIFS) program in Indonesia was replaced by providing education about anaemia through online media (Soewondo et al. 2020), which may not have been as effective. Similarly, it has been reported from South African countries that risk of anaemia among female adolescents increased during the COVID‐19 pandemic due to irregular use of IFA tablets. Moreover, female adolescents from low‐income families were particularly vulnerable due to reduced access to healthy foods, increased food insecurity, uncertainty in finding work, and lower physical activity levels (Dorsamy et al. 2022). Interestingly, our data showed improved food access during the pandemic (Table 1), possibly due to government assistance consisting of the provision of cash money or foods (typically rice, noodles and oil), which may have helped to protect households against food insecurity(Indonesian Ministry of Finance, et al. 2022). However, this is not a guarantee for the purchase and consumption of nutrient‐dense foods. In addition, risky food consumption did not emerge as a significant contributor to haemoglobin concentration in our analysis, suggesting that other factors, such as dietary quality and nutrient density, may have played a more prominent role.

To our knowledge, the current study is the first to longitudinally examine the association between sleep quality, depression, and haemoglobin concentration among adolescents in a low‐ and middle‐income country (LMIC), specifically in Indonesia. By conducting assessments at two time points 1 year apart, this study provides evidence of the temporal relationship between these factors, enabling inferences regarding the direction of causality. A key strength of our study is the use of LCA to classify adolescents into groups based on shared patterns of sleep quality and depression. This approach moves beyond traditional categorical diagnostic constructs, allowing for a more nuanced understanding of the interplay between these variables.

However, we also acknowledge some limitations of the study: the number of adolescents in group D and E were relatively small, which may have limited the power to detect associations. In addition, there may be residual confounding from factors that were not considered in the analysis, such as dietary intake, morbidity, and inflammation (Neumann et al. 2021). As the present study was conducted in rural areas, we acknowledge that the findings may not be generalisable to urban setting. Self‐reported sleep quality and depression can be biased, although the questionnaires had been adapted, translated, and validated for adolescents in the Indonesian setting. Additionally, potential misclassification in the LCA due to measurement bias should be considered, particularly since key indicators were self‐reported. A longer prospective study would be warranted to further explore the longitudinal relationship between sleep quality, depression, and risk of anaemia in adolescents.

In conclusion, we demonstrated that adolescents with moderate‐to‐good sleep quality and low‐to‐moderate risk of depression had significantly lower haemoglobin concentrations compared to their peers with good sleep quality and low risk of depression. Our results advance the field by identifying previously unexplored pathways through which psychosocial factors may influence anaemia risk. Practically, the findings suggest that integrating mental health and sleep quality interventions, such as promoting healthy sleeping habits, managing stress, and providing mental health support, into anaemia prevention programs could enhance their effectiveness. Such an integrated approach has the potential to improve not only haemoglobin concentrations but also overall adolescent well‐being. These findings can help shaping future school‐ and community‐based programs to reduce anaemia in adolescents by combining nutritional support with interventions that improve mental health and sleep quality.

## Author Contributions

All authors had an essential role in formulation of the research questions, M.A. wrote the first draft of the paper and analysed the data, all authors were involved in interpretation of the data and revision of the manuscript. All authors have read and approved the final paper.

## Ethics Statement

The study proposal and protocols have been approved by the ethical committee of the Faculty of Medicine, Public Health, and Nursing, Universitas Gadjah Mada (UGM). The first ethical approval was granted in 2020 (Letter number: KE/FK/0039/EC/2020), but due to COVID‐19 pandemic the study was postponed to 2021 with granted ethical approval in 2021 (Letter number: KE/FK/0009/EC/2021). The study was also permitted by a local permit of the District Level office (Letter number: 070/00189) and by the District Health Office (DHO).

## Conflicts of Interest

The authors declare no conflicts of interest.

## Public Involvement

The community was involved at several stages of the study. We received input from local stakeholders who lived in selected study sites of which they provided data for the sampling method and some considerations related to the current situation of COVID‐19 pandemic. We followed pandemic related rules to conduct the interview. We intend to disseminate the main results to respondents and are still developing of an appropriate method of dissemination to stakeholders and health workers.

## Supporting information

Supporting Information S1

## Data Availability

The datasets are not publicly available due to ethical restrictions to minimize participant risk and maximize privacy and confidentiality as much as possible. However, anonymized data are available from the corresponding author upon reasonable request.
