# Supplementary material for: Sleep Quality, Depression, and the Risk of Anaemia in Adolescents Aged 10–19 Years During One Year of the COVID‐19 Pandemic in Indonesia
Source: Stress Health. 2025 May 3;41(3):e70046. doi: 10.1002/smi.70046 (PMC12049092; doi:10.1002/smi.70046)
Supplement: Supplementary file 1 — Supporting Information S1 [file SMI-41-e70046-s001.docx]

**Supplementary Table 1.** PSQI and K10 questionnaires

| Instructions: The following questions relate to your usual sleep habits during the past month only, Your answers should indicate the most accurate reply for the majority of days and nights in the past month, Please answer all questions, During the past month, *Petunjuk: Pertanyaan-pertanyaan berikut ini berkaitan dengan kebiasaan tidur Anda yang biasa selama sebulan terakhir, Jawaban Anda sebaiknya mewakili tidur sebagian besar siang dan malam dalam sebulan terakhir, Mohon jawab semua pertanyaan, Selama sebulan terakhir,*   1. When have you usually gone to bed? *Kapan biasanya Anda tidur?* 2. How long (in minutes) has it taken you to fall asleep each night? *Berapa lama (dalam menit) Anda biasanya butuhkan untuk tertidur setiap malam?* 3. When have you usually gotten up in the morning? *Kapan biasanya Anda bangun di pagi hari?* 4. How many hours of actual sleep do you get at night? (This may be different than the number of hours you spend in bed) *Berapa jam Anda tidur di malam hari? (Ini mungkin berbeda dari jumlah jam yang Anda habiskan di tempat tidur)* |
| --- |

| 1. During the past month, how often have you had trouble sleeping because you,,,   *Selama sebulan terakhir, seberapa sering Anda sulit tidur karena ,,,*  *Answer:* **Not during the past month** *Tidak selama sebulan terakhir,* **Less than once a week (1)** *Kurang dari sekali seminggu (1)*; **Once or twice a week (2);** *Sekali atau dua kali seminggu (2)* **Three or more times week (3)** *Tiga kali atau lebih dalam seminggu (3)* |
| --- |
| 1. Cannot get to sleep within 30 minutes *Tidak bisa tidur dalam 30 menit* |
| 1. Wake up in the middle of the night or early morning *Bangun di tengah malam atau dini hari* |
| 1. Have to get up to use the bathroom *Harus bangun untuk ke kamar mandi* |
| 1. Cannot breathe comfortably *Tidak bisa bernapas dengan nyaman* |
| 1. Cough or snore loudly *Merasa terlalu dingin* |
| 1. Feel too cold *Merasa terlalu dingin* |
| 1. Feel too hot *Merasa terlalu panas* |
| 1. Have bad dreams *Mimpi buruk* |
| 1. Have pain *Sakit* |
| 1. Other reason(s), please describe, including how often you have had trouble sleeping because of this reason(s): *Alasan lain, harap jelaskan, termasuk seberapa sering Anda sulit tidur karena alasan ini:* ,,,,,,,,,,,,,,,,,,,,,,,,,,,,,,,,,,,,,,,,,,,,,,,,, |
| 1. During the past month, how often have you taken medicine (prescribed or “over the counter”) to help you sleep? *Selama sebulan terakhir, seberapa sering Anda minum obat (dengan resep atau beli sendiri untuk membuat Anda tidur?* |
| 1. During the past month, how often have you had trouble staying awake while driving, eating meals, or engaging in social activity? *Selama sebulan terakhir, seberapa sering Anda mengalami kesulitan untuk tetap terjaga saat mengemudi, makan, atau melakukan aktivitas sosial?* |
| 1. During the past month, how much of a problem has it been for you to keep up enthusiasm to get things done? *Selama sebulan terakhir, seberapa besar masalah yang Anda alami dalam mempertahankan antusiasme untuk menyelesaikan sesuatu?* |
| 1. During the past month, how would you rate your sleep quality overall?   *Selama sebulan terakhir, bagaimana Anda menilai kualitas tidur Anda secara keseluruhan?* |

**The Kessler Psychological Distress Scale English and Bahasa Version (K10)**

| Anxiety and depression checklist (K10)  These questions relate to how you've been feeling over the past four weeks, Give an answer to each question that best reflects your thoughts, feelings and behaviour, **None of the time, A little of the time, Some of the time, Most of the time, or All of the time**  *Berikut adalah pertanyaan-pertanyaan mengenai bagaimana Anda merasakan berbagai situasi di bawah ini dalam kurun waktu 4 minggu terakhir, Pilih jawaban yang paling tepat untuk menggambarkan seberapa sering kamu mengalaminya dengan memberikan tanda: Tidak pernah, jarang, kadang-kadangm sering, atau selalu* |
| --- |
| About *tentang*: |
| 1. how often did you feel tired for no good reason? *kira-kira seberapa sering Anda merasa lelah tanpa alasan yang jelas?* |
| 1. how often did you feel nervous? *kira-kira seberapa sering anda merasa gugup?* |
| 1. About how often did you feel so nervous that nothing could calm you down? *kira-kira seberapa sering anda merasa gugup sehingga tidak ada yang dapat menenangkan diri anda?* |
| 1. About how often did you feel hopeless? *kira-kira seberapa sering anda merasa putus asa?* |
| 1. About how often did you feel restless or fidgety? *kira-kira seberapa sering anda merasa gelisah atau tidak nyaman?* |
| 1. About how often did you feel so restless you could not sit still? *Kira-kira seberapa sering Anda merasa sangat gelisah sehingga tidak dapat duduk tenang?* |
| 1. About how often did you feel depressed? *kira-kira seberapa sering anda merasa depresi/sedih?* |
| 1. About how often did you feel that everything was an effort?   *kira-kira seberapa sering anda merasa bahwa segala sesuatu memerlukan usaha berat?* |
| 1. About how often did you feel so sad that nothing could cheer you up?   *kira-kira seberapa sering anda merasa sangat sedih dan tidak ada yang dapat membuat anda ceria?* |
| 1. About how often did you feel worthless?   *kira-kira seberapa sering anda merasa tidak berarti?* |

**Supplementary Table 2.** Variable definition, scores, and survey question

*Socioeconomic and characteristic*

| **Variable** | **Definition** | **Question/measurement** | **Answering options** | **Classification/ analysis** |
| --- | --- | --- | --- | --- |
| **Adolescent variables** |  |  |  |  |
| Adolescent’s Occupation | Occupation refers to activities in which adolescents engage when pursuing their pleasure daily | Scale | 1. No work 2. School 3. Civil servant, army, police, governmental organization 4. Non-governmental organization 5. Entrepreneur 6. Farmer 7. Fisherman 8. Driver/ housekeeper   Other | 1: Unemployed (if 1)  2: Student (if 2)  3: Work (paid or unpaid) (if 3-9) |
| Smoking Status | Smoking status is a recoded variable based on a question about cigarette smoking | Smoking status | 1: Yes  2: No | 1: Yes  0: No |
| Alcohol consumption | Alcohol consumption refers to frequency of consumption of alcohol on daily and weekly basis | Alcohol consumption in the past month | 1: Yes  2: No | 1: Yes  0: No |
|  |  | Total days of alcohol consumption in the past month | …, Days | Continuous data (number of days) |
| Nutritional status | Nutritional status is adolescent’s weight status indicated by BMI-for age-z-score | Height (cm) and weight (kg) to calculate BMI-for-age Z-score | Scale | 1: < -3SD (Severe thinness)  2: ≥ -3 SD ≤ -2 SD (Thinness)  3: >-2 SD to ≤1 SD (Normal)  4: >1 SD to ≤2 SD (Overweight)  5: >2 SD (Obese) |
| Physical activity | Physical activity is any bodily movement resulting in energy expenditure, reported by recall of the individual’s movement | PAQ- A |  |  |
| Anaemia | Anaemia is a condition in which the number of red blood cells or haemoglobin concentration is lower than normal | Hemocue / Haemoglobin (g/dL) | Based on measurement using Hemocue, Hb is in g/dL | 3 new variables:   1. **Anaemia and non-anaemia**   Age 10-11 years:  No-anaemia: 11,5 or higher  Anaemia: lower than 11,5  Age 12-4 years  No-anaemia: 12 or higher  Anaemia: lower than 12  Girls age 15 years or above  No-anaemia: 12 or higher  Anaemia: lower than 12  Boys age 15 years or above  No-anaemia: 13 or higher  Anaemia: lower than 13   1. **Anaemia classification**   Age 10-11 years:  No-anaemia: 11,5 or higher  Mild anaemia: 11-11,4  Moderate anaemia: 8-10,9  Severe anaemia: lower than 8  Age 12-4 years  No-anaemia: 12 or higher  Mild anaemia: 11-11,9  Moderate anaemia: 8-10,9  Severe anaemia: lower than 8  Girls aged 15 years or above  No-anaemia: 12 or higher  Mild anaemia: 11-11,9  Moderate anaemia: 8-10,9  Severe anaemia: lower than 8  Boys aged 15 years or above  No-anaemia: 13 or higher  Mild anaemia: 11-12,9l  Moderate anaemia: 8-10,9  Severe anaemia: lower than 8   1. **Continuous data Hb** |
| Anaemia (altitude adjustment) | Anaemia level with taking into account of altitude (Hb correction) | Hemocue / Haemoglobin (g/dL) | Based on measurement using Hemocue, Hb is in g/dL | ***Hb correction = –0,032 (altitude × 0,0032808) + 0,022 (altitude × 0,0032808)^2^***  The classification is the same the previous one, |
|  |  |  |  |  |
| **Household variables** |  |  |  |  |
| Parental education | Parental education is defined as the highest education level of parents attained | Household questionnaire: *‘What is your (father/ mother) highest? last education’* | 1. Never attended school 2. Elementary school (not completed) 3. Elementary school (completed) 4. Middle school (completed) 5. Senior high school (completed) 6. Diploma (completed) 7. University (completed) | 1: No education (if 1, 2)  2: Elementary school (if 3,4)  3: Senior high school (if 5)  4: Diploma or higher (if 6,7) |
| Parental occupation | Parental occupation is defined as activities in which parents engage on a daily basis to have income | Household questionnaire: *‘What is your (father/ mother) occupation?’* | 1. No work 2. School 3. Civil servant, army, police, governmental organization 4. Non-governmental organization 5. Entrepreneur 6. Farmer 7. Fisher 8. Driver/ housekeeper   Other | 1: Unemployed (if 1, 2)  2: Unsecured job (if 5, 6, 7, 9)  3: Secured job (if 3, 4, 8) |
| Number of household members | Number of household members is defined as the number of people living in the same building, not considering the household registration | Household questionnaire*: ‘How many people live in this house/ building (not based on household)?’* | Total number of people | 1: ≤ 5 people  2: > 5 people |
| Wealth index | Wealth index measured by ownership of the goods and expenses for food and non-food, using 10 items of questionnaire and classified using on Central Bureau of Statistics (BPS) Indonesia | BPS questionnaire | Scoring from 0-10 | 1: Poor Household  2: Not a poor household |
| Household income | All income from all family members for food and non-food expenses | Household questionnaire: | 1. < Rp 1,000,000 2. Rp 1,000,000 - Rp 3,000,000 3. Rp 3,000,000 - Rp 5,000,000 4. Rp 5,000,000 - Rp 7,000,000 5. > Rp 7,000,000 | Total Household income:   1. < Rp 3,000,000 2. Rp 3,000,000 - Rp 5,000,000 3. > Rp 5,000,000 |
| Household insurance | Insurance ownership of household either given by government, self-payment/ out of pocket, or both | Household questionnaire:  *Type of family health insurance* | 1. National Health Insurance (BPJS)- full covered by government 2. National Health Insurance (BPJS)-self payment 3. Jamkesos 4. Jamkesda | 1. Government insurance 2. Private insurance 3. Combination |
| Access to health care services | Accessibility of health care services based on respondent’s knowledge | Household questionnaire:  *Do household members know of the nearest hospital / Puskesmas / Pustu / Posyandu?*  *How long it takes to reach the facility?* | 1. In the city 2. In closest city 3. Nothing 4. Do not know | 1: Accessible  2: Not accessible |
| Demographic area | Demographic area was divided into rural and urban, assessed using classification of Indonesia’s Bureau of Statistics, which is based on population density, percentage of agricultural households, and presence/access to facilities | BPS Classification | 1: Rural area  2: Urban area | 1: Rural area  2: Urban area |

**Pubertal development and body image**

| **Variable** | **Definition** | **Question/measurement** | **Answering options** | **Classification** |
| --- | --- | --- | --- | --- |
| Pubertal development | Puberty is broadly defined as the time at which a child develops secondary sexual characteristics and reproductive function, | The Self-Rating Scale for Pubertal Development | Boys and girls (5 questions)  1: has not yet begun  2: has barely started  3: is definitely underway  4: seems completed  0: I don’t know  Especially for girls’ question about menarche:  4: Yes  1: No | **Boys:**  Prepubertal = 3  Early Pubertal = 4 or 5 (no 3-point responses)  Mid-pubertal = 6, 7, or 8 (no 4-points)  Late pubertal = 9-11  Post-pubertal = 12  **Girls:**  Prepubertal = 2 and no menarche  Early Puberty = 3 and no menarche  Mid-pubertal = > 3 and no menarche  Late Puberty = <= 7 and menarche  Post-pubertal = 8 and menarche, |
|  |  | *Self-reported picture* | **Pubic Hair Scale (both males and females)**  Stage 1, Stage 2, Stage 3, Stage 4, Stage 5  **Female Breast Development Scale**  Stage 1, Stage 2, Stage 3, Stage 4, Stage 5  **Male External Genitalia Scale**  Stage 1, Stage 2, Stage 3, Stage 4, Stage 5 | **3 generate variables in each development indicators**  ***A (Classification I)***  1: pre-pubertal (stage 1)  2: early pubertal  (stages 2–3)  3: late pubertal (stages 4–5)  ***B (Classification II)***  1: pre pubertal (stage 1)  2: Pubertal (stage 4-5)  ***C, Continuous data*** |

Mental health and well-being

| **Variable** | **Definition** | **Question/measurement** | **Answering options** | **Classification** |
| --- | --- | --- | --- | --- |
| Anxiety and depression | Measurement of distress based on questions about anxiety and depressive symptoms that a person has experienced in the most recent 4-week period, | K-10: the simple checklist asks you to reflect on your feelings over the past four weeks,  ***Note:***  The maximum score is 50 indicating severe distress, the minimum score is 10 indicating no distress,  Questions 3 and 6 are not asked if the preceding question was ‘none of the time’ in which case questions 3 and 6 would automatically receive a score of one, | 1: All of the time  2: most of the time  3: some of the time  4: a little of the time  5: none of the time  ***Note:***  The maximum score is 50 indicating severe distress, the minimum score is 10 indicating no distress,  Questions 3 and 6 are not asked if the preceding question was ‘none of the time’ in which case questions 3 and 6 would automatically receive a score of one, | **Total Score**  ***Scoring:***  1: All of the time, score 5  2: most of the time, score 4  3: some of the time, score 3  4: a little of the time, score 2  5: none of the time, score 1  ***Note:***  The maximum score is 50 indicating severe distress, the minimum score is 10 indicating no distress,  Questions 3 and 6 are not asked if the preceding question was ‘none of the time’ in which case questions 3 and 6 would automatically receive a score of one, |
| Eating disorders | An eating disorder is a mental disorder defined by abnormal eating habits that negatively affect a person's physical and/or mental health | EAT-26 questionnaire with 26 items of questions | 1: Always  2: Usually  3: Often  4: Sometimes  5: Rarely  6: Never | **Classification:**  0: No (total score<20)  1: have eating disorder (total score>20)  **Continues data (Total score)**  ***Note:***  For all the items, except no 26, for each of the answer assign the score as following:  Always =3 Usually =2 Often =1, Sometimes =0 Rarely =0  Never =0  For item no 26 assign the score as following:  Always =0 Usually =0 Often =0 Sometimes =1 Rarely =2  Never =3 |
| Sleep quality index | a self-report questionnaire that assesses sleep quality over a 1-month time interval, | The Pittsburgh Sleep Quality Index (PSQI) | **Question C22-C24:**  1: Not during the past month (0)  2: Less than once a week (1)  3: Once or twice a week (2)  4: Three or more times a week (3)  **Questions C25:**  1: Very good  2: Fairly good  3: Fairly bad  4: Very bad | **Question C22-C24:**  1: Not during the past month (score 0)  2: Less than once a week (score 1)  3: Once or twice a week (score 2)  4: Three or more times a week (score 3)  **Question C25:**  1: Very good, score 0  2: Fairly good, score 1  3: Fairly bad, score 2  4: Very bad, score 3  **Total Scoring**  *Component 1:*  #C25 Score  *Component 2:*  #C19 Score (<15min (0), 16-30min (1), 31-60 min (2), >60min (3)) + #C21a Score (if sum is equal 0=0; 1-2=1; 3-4=2; 5-6=3)  *Component 3:*  #C20 Score (>7(0), 6-7 (1), 5-6 (2), <5 (3)  *Component 4:*  (total # of hours asleep) / (total # of hours in bed) x 100 >85%=0, 75%-84%=1, 65%-74%=2, <65%=3  *Component 5:*  # sum of scores C21b to 5j (0=0; 1-9=1; 10-18=2; 19-27=3)  *Component 6:*  #6 Score  *Component 7:*  #C23 Score + #8C24 score (0=0; 1-2=1; 3-4=2; 5-6=3)  ***Total score: Add the seven component scores together***  ***Classification:***  0: normal sleep (if total score<5)  1: poor sleep (if total score equal or higher than 5) |

Food consumption

| **Variable** | **Definition** | **Question/measurement** | **Answering options** | **Classification** |
| --- | --- | --- | --- | --- |
| Risk consumption | Unhealthy food consumption refers to consumption of salty food, high-fat food, and soft drinks |  | 1. >1x /day 2. 1x /day 3. 3 – 6x /week 4. 1 – 2x /week 5. <3x /month 6. Never 7. Not answered | **Continuous data (total score)**  Selected answer (score =1)  Unselected answer (score =0) |
| Food consumption before and after pandemic | Frequently Foods consumed before and during the quarantine/pandemic | DDS/scale | Yes: 1 No: 0 | Tertiles of the each frequently consumed food before pandemic (6 months before), and during Pandemic (within 6 months) |
| Diet Diversity Score | Total score of several food groups consumed during the las 24 hours | MMD-W  Foods consumed will be assigned into 10 different food groups   - Starchy staples - Dark green leafy vegetables - Vitamin A-rich fruits, tubers & vegetables - Other vegetables - Other fruits - Flesh and organ meats - Eggs - Fish - Legumes, nuts, seeds - Milk products   Reference:  Minimum Dietary Diversity for Women, A guide to measurement  FAO, 2016  https://www.mdpi.com/2072-6643/12/8/2230/htm | **Starcy staples:**   - Rice, cassava, potato, sweet potato, bread   **Dark-green leafy vegetables**:   - Spinach, daun kacang panjang (lembayung), daun ubi   **Vitamin A-rich fruits & vegetables**   - Carrot, red guava, orange-sweet potato, papaya (ripe), mango (ripe)   **Other vegetables**   - Cucumber, cauliflower, green beans, bitter melon etc   **Other fruits**   - Banana, orange,   **Flesh and organ meat**:   - Chicken, beef, pigeon, duck, liver,   **Eggs**   - Chicken, duck, quail eggs   **Fish**   - Seafoods, prawn, gurameh and other fish   **Legumes, nuts, seeds**   - Tahu, tempe, mung bean, adzuki bean, kidney bean,   **Milk products:**   - Milk, cheese, yoghurt, peanuts | 1. Identify each food consumed by adolescent and assigned into food group 1 – 10 2. Score 1: if they consumed at least 1 food item of each food group, at least 1 tablespoon of foods 3. Sum the food groups consumed by the adolescent   **DDS < 5: inadequate DD**  **DDS ≥ 5: adequate DD** |

**Supplementary Table 3.** LCA cluster models’ fit and simplicity statistics

|  |  | **LL** | **BIC** | **AIC** | **Npar** | **L²** | **df** | **p-value** | **Max,BVR** |
| --- | --- | --- | --- | --- | --- | --- | --- | --- | --- |
| Model1 | 1-Cluster | -6540,95 | 13436,83 | 13197,9 | 58 | 7780,115 | 397 | <0.001 | 43,3921 |
| Model2 | 2-Cluster | -5868,88 | 12453,75 | 11971,77 | 117 | 6435,984 | 338 | <0.001 | 13,9479 |
| Model3 | 3-Cluster | -5695,85 | 12468,73 | 11743,7 | 176 | 6089,915 | 279 | <0.001 | 4,2782 |
| Model4 | 4-Cluster | -5593,67 | 12625,42 | 11657,35 | 235 | 5885,563 | 220 | <0.001 | 4,3315 |
| Model5 | 5-Cluster | -5522,99 | 12845,1 | 11633,97 | 294 | 5744,191 | 161 | <0.001 | 4,1527 |
| Model 6 | 6-Cluster | -5467,25 | 13094,67 | 11640,5 | 353 | 5632,712 | 102 | <0.001 | 4,3726 |
| Model 7 | 7-Cluster | -5428,14 | 13377,49 | 11680,27 | 412 | 5554,488 | 43 | <0.001 | 3,4612 |
| Model 5+ direct effects* | 5-Cluster | -5499,74 | 12853,69 | 11605,49 | 303 | 5697,702 | 152 | <0.001 | 3,495 |

^*^Direct effects from component of sleep duration (component 3) and sleep medication (component 6). Bold values designated the selected model with the best balance between model fit and model simplicity based on Bayesian information Criterion (BIC) and Akaike Information Criterion (AIC), The lower BIC and AIC, the better the balance

**Supplementary Table 4.** Characteristics comparison of exclude and included data from analysis^1^

| Characteristics | Excluded case | Included Cases | P-value^1^ |
| --- | --- | --- | --- |
|  | N= 124 | N=452 |  |
| Adolescent characteristics |  |  |  |
| Sex |  |  |  |
| Boys | 64 (51.6) | 222 (49.1) | 0.724 |
| Girls | 60 (48.4) | 230 (50.9) | 0.730 |
| Age group |  |  |  |
| 10 - ≤ 13y | 19 (15.3) | 130 (28.8) | 0.216 |
| >13 - ≤ 16 y | 28 (22.6) | 169 (37.4) | 0.129-1 |
| >16 - ≤ 18 y | 61 (49.2) | 133 (29.4) | 0.057 |
| >18 - 19 y | 16 (12.9) | 20 (4.4) | 0.355 |
| Adolescent occupation |  |  |  |
| Unemployed | 32 (25.8) | 38 (8.4) | 0.059 |
| Student | 84 (67.7) | 401 (88.7) | 0.001 |
| Work | 8 (6.5) | 13 (2.9) | 0.692 |
| Physical activity |  |  |  |
| Low | 37 (29.8) | 150 (33.2) | 0.692 |
| Moderate | 53 (42.7) | (181 (40.0) | 0.724 |
| High | 34 (27.5) | 121 (26.8) | 0.935 |
| Nutritional status (BMI z-score) |  |  |  |
| Severely thin | 3 (2.4) | 8 (1.8) | 0.949 |
| Thin | 9 (7.3) | 43 (9.6) | 0.828 |
| Normal | 92 (74.2) | 300 (66.8) | 0.181 |
| Overweight | 11 (8.8) | 62 (13.8) | 0.189 |
| Obese | 9 (7.3) | 36 (8.0) | 0.944 |
| Disabilities |  |  |  |
| None | 51 (41.2) | 198 (43.8) | 0.738 |
| Low | 41 (33.0) | 107 (23.7) | 0.250 |
| Moderate | 252 (20.2) | 121 (26.8) | 0.152 |
| Severe/Very Severe | 7 (5.6) | 26 (5.7) | 0.991 |
| Household characteristic |  |  |  |
| Paternal education |  |  |  |
| No education | 10 (8.3) | 35 (7.8) | 0.958 |
| Elementary school | 75 (60.2) | 271 (60.2) | 1.000 |
| Senior high school | 35 (28.2) | 125 (27.8) | 0.962 |
| University | 4 (3.3) | 19 (4.2) | 0.933 |
| Maternal education |  |  |  |
| No education | 9 (7.3) | 36 (8.1) | 0.936 |
| Elementary school | 91 (73.4) | 285 (64.3) | 0.109 |
| Senior high school | 18 (14.5) | 95 (21.4) | 0.504 |
| University | 6 (4.8) | 27 (6.1) | 0.902 |
| Paternal occupation |  |  |  |
| Unemployed | 6 (4.8) | 1 (0.2) | 0.830 |
| Unsecured job | 64 (51.6) | 195 (45.4) | 0.388 |
| Secured Job | 54 (43.6) | 234 (54.4) | 0.152 |
| Maternal occupation |  |  |  |
| Unemployed | 41 (33.1) | 146 (35.2) | 0.802 |
| Unsecured job | 58 (46.8) | 201 (48.4) | 0.829 |
| Secured job | 25 (20.1) | 68 (16.4) | 0.676 |
| Number of household members > 5 people | 23 (18.6) | 95 (21.0) | 0.798 |
| Household income |  |  |  |
| Low | 102 (82.3) | 375 (82.9) | 0.886 |
| Moderate | 16 (12.9) | 62 (13.8) | 0.925 |
| High | 6 (4.8) | 15 (3.3) | 0.869 |

^1^Values are n (%) for categorical data
